# iPathCase^KEGG^: An iPad interface for KEGG metabolic pathways

**DOI:** 10.1186/2047-2501-1-4

**Published:** 2013-01-10

**Authors:** Stephen R Johnson, Xinjian Qi, A Ercument Cicek, Gultekin Ozsoyoglu

**Affiliations:** Electrical Engineering and Computer Science Department, Case Western Reserve University, Cleveland, OH USA

**Keywords:** KEGG, iPad, App, PathCase, Metabolic networks, Pathways

## Abstract

**Background:**

Kyoto Encyclopedia of Genes and Genomes (KEGG) is an online and integrated molecular database for several organisms. KEGG has been a highly useful site, helping domain scientists understand, research, study, and teach metabolisms by linking sequenced genomes to higher level systematic functions. KEGG databases are accessible through the web pages of the system, but the capabilities of the web interface are limited. Third party systems have been built over the KEGG data to provide extensive functionalities. However, there have been no attempts towards providing a tablet interface for KEGG data. Recognizing the rise of mobile technologies and the importance of tablets in education, this paper presents the design and implementation of iPathCase^KEGG^, an iPad interface for KEGG data, which is empowered with multiple browsing and visualization capabilities.

**Results:**

iPathCase^KEGG^ has been implemented and is available, free of charge, in the Apple App Store (locatable by searching for “Pathcase” in the app store). The application provides browsing and interactive visualization functionalities on the KEGG data. Users can pick pathways, visualize them, and see detail pages of reactions and molecules using the multi-touch interface of iPad.

**Conclusions:**

iPathCase^KEGG^ provides a mobile interface to access KEGG data. Interactive visualization and browsing functionalities let users to interact with the data in multiple ways. As the importance of tablets and their usage in research education continue to rise, we think iPathCase^KEGG^ will be a useful tool for life science instructors and researchers.

## Background

Kyoto Encyclopedia of Genes and Genomes (KEGG) is one of the well-known biological knowledge bases, that links genomic information to high-level functions of the cell, for several organisms [1, 2]. KEGG, among others, provides the association between sequenced genomes and their correspondences in metabolic networks and biochemical pathways [1, 2]. Although the KEGG system has added over time, new tools and additional databases such as KEGG Brite, the core of the system is centered on KEGG Pathways, which specify the sets of reactions that participate in biochemical pathways for different organisms. KEGG Genes and KEGG Ligand databases specify information about the associated genes and the chemicals/molecules for KEGG Pathways, respectively. Through a web interface, KEGG provides maps of metabolic pathways for visualization purposes. KEGG pathways data is made available at an FTP site, and web services for third party applications are provided as an API.

PathCase^KEGG^ system is one of the PathCase family of systems, with a PathCase^KEGG^ database, populated from KEGG data. PathCase^KEGG^ system provides, over the web, additional browsing, querying, visualization, and analysis functionalities for KEGG data, such as interactive visualization [3]. And, PathCase-SB, another PathCase system, integrates KEGG data with data from other data sources [4, 5] such as BioModels [6, 7]. Both PathCase systems provide hierarchical browsing; a query interface and a JAVA applet-based interactive visualization of pathways.

In this paper, we describe iPathCase^KEGG^ which is an iPad-based system with a mobile interface that accesses PathCase^KEGG^, KEGG Pathways, and ENZYME databases, and provides browsing and interactive visualization functionalities on the accessed data. To the best of our knowledge, as of August 2012, there have been no attempts, with the exception of SimGene [8], towards providing a mobile interface for browsing and visualizing KEGG pathways. SimGene, designed for iPhone (not for iPad), is a gene-related information searching tool, and does not focus on pathway browsing or visualization. More specifically, given a gene symbol, SimGene searches many databases (Simbiot, Ensembl, NCBI, Gene Ontology, KEGG Pathways, PubMed, Genomic Variations and other databases) for more than 30 species, and provides annotated information about the specified gene.

iPad has been a revolutionizing mobile device with the multi-touch based interface. Apple has sold 9.25 million iPads only in the fiscal 2011 third quarter [9]. With the releases of similar devices by other brands, tablets are becoming widespread. Ease of use with familiar gestures and mobility of tablets have contributed to their success. However, websites designed for computers are not optimized for mobile devices. Therefore, companies release their own apps, or redirect users to mobile versions of their web pages. To the best of our knowledge, there is no such app or mobile version for the KEGG web site.

Tablets also have started to attract attention in education. For instance, Apple has made an effort to revolutionize text books [10], and countries distribute tablets to school children (such as Turkey [11]). We think that KEGG is an invaluable resource for life sciences education, and there is a need for a mobile interface for instructors and researchers to access KEGG data. In this paper, we present iPathCase^KEGG^, an iPad application to interface with KEGG data. The system comes with the following functionalities:

- Interactive visualization of metabolic pathways,
- Access to the KEGG web page regarding a pathway within the app,
- Highlighting a node and close proximity,
- Highlighting organism-specific subsets of reactions,
- Enzyme details pane from ENZYME Database for a selected reaction through EC (Enzyme Commission) Numbers [12].

iPathCase^KEGG^ has been implemented, extensively tested for iPad 2, and is freely available in Apple App Store.

### Overview of KEGG

As of July 23, 2012, KEGG consists of seventeen databases: KEGG ORTHOLOGY, GENOME , GENES, DGENES and SSDB in genomic information, KEGG COMPOUND, GLYCAN, REACTION, RPAIR, RCLASS and ENZYME in chemical information, KEGG PATHWAY, BRITE, MODULE, DISEASE, DRUG and ENVIRON in systems information. The most unique data object in KEGG is the molecular networks -- molecular interaction, reaction and relation networks representing systemic functions of the cell and the organism. Systemic functions capture experimental knowledge from literature, which are organized in three forms: Pathway maps - in KEGG PATHWAY, Hierarchical list (ontology) - in KEGG BRITE, Membership list - in KEGG MODULE and KEGG DISEASE. These databases constitute the reference knowledge base for biological interpretation of genomes and high-throughput molecular datasets through the process of KEGG mapping, which is a process to map elementary datasets (genes, proteins, small molecules, etc.) to network datasets (KEGG pathway maps, BRITE functional hierarchies, and KEGG modules). Currently there are four types of mapping operations available in KEGG: pathway mapping, brite mapping, module mapping and taxonomy mapping, which may involve molecular or non-molecular datasets (orthologs, modules, and organisms) and the network dataset (taxonomic tree). While the main focus of KEGG has been the molecular network, different types of data and knowledge, such as disease genes and drug targets, are also integrated as part of the KEGG molecular networks to support translational bioinformatics.

### Overview of PathCase^KEGG^

*PathCase*^*KEGG*^ is a web-based system to store, browse, query, visualize and analyze KEGG metabolic pathways at different levels of genetic, molecular, biochemical and organismal detail. It integrates (i) browsing interface to access different pathway information and pathway-related information (processes, molecular entities, genes), (ii) an interactive client-side visualization tool for metabolic pathways, with powerful visualization capabilities, and with integrated gene and organism viewers, (iii) three distinct querying capabilities: keyword search function for pathways, processes, organisms, basic molecules, proteins and genes, an advanced querying interface for computer savvy users, and built-in queries for ease of use, that can be issued directly from pathway visualizations, (iv) pathway functionality analysis tools, such as the gene ontology viewer, and (v) pathway and process export and import capabilities.

### Overview of iPad interface

iPad 2 (hereafter simply “iPad”) is a device about 7.3x9.5x0.3 inches with a 1024x768 pixel touch screen [13]. The touch screen can detect more than one human finger at a time, allowing for actions such as zooming to be accomplished with natural gestures using two or more fingers. iPad runs iOS, a UNIX-based operating system which presents one full-screen application at a time and provides multithreading, persistent storage, wireless network access, and other services. This section describes only those iPad-specific user interface concepts that iPathCase^KEGG^ uses.

Unlike traditional computer displays, the iPad is designed to be held in any orientation, with the display’s contents rotating and resizing to fit the users perspective. There are four possible orientations, but the shortcuts “landscape” and “portrait“ suffice for describing and implementing most interfaces [13].

The most basic component of an iPad interface is the “view”. A view is a rectangular region that is capable of displaying content, receiving events such as gestures and device orientation changes, and delegating these tasks to “subviews”. Views can contain buttons, toolbars, lists, other views, and arbitrary graphical content. “Nested views” consist of a view hierarchy.

A “button” is a view that renders itself in enabled, disabled, and pressed states, and invokes some action when a touch is released inside its bounds.

“Toolbars” are views that can be inserted at the top or bottom of another view. They typically contain buttons, text labels, progress indicators, and more.

A “scroll view” contains another, larger view that the user can move around with one or two fingers. For example, one might be used to display a photo that is larger than the screen. Scroll views can be dragged around with one finger, or zoomed out by “pinching two fingers” together.

“Tapping twice” in the same location typically zooms in on the tapped location.

One common use for the scroll view is in a table view, used to display lists of content. The user can scroll up and down the list and tap a row to invoke some action. Some types of views exist only to contain other views. One view of this type is the “popover”. A popover appears temporarily on the screen, allowing the user to perform some quick interaction before dismissing it by tapping outside its bounds. The popover is a key component in the master-detail interface, a common arrangement of views in iPad applications.

**Figure 1:**
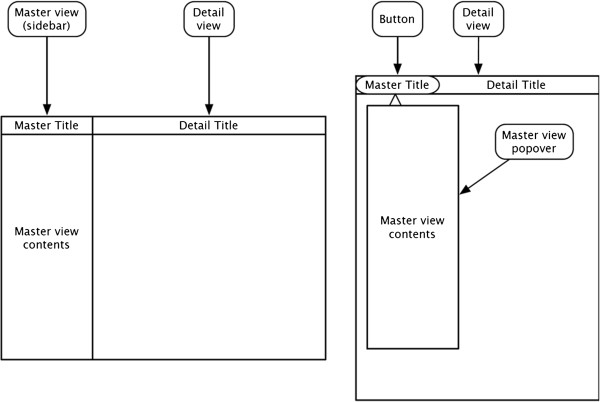
**Master-detail interface in landscape and portrait orientations.** Figure shows the organization of a typical view of the application on iPad. The thin layer on the left is called the master view, which contains the data to be navigated. The right view is the detail view, into which the selected data is loaded.

A diagram of the master-detail interface is shown in Figure 1. When the device is in a landscape orientation, a “thin view” is shown to the left of a wide view. The “thin view”, called the master view or sidebar, contains content for navigating the application’s data, such as a table view of email messages. The “wide view”, called the detail view or content view, shows the selection made in the master view. When the device is in a landscape orientation, the detail view is resized to fill the screen. A button on the left side of the top toolbar displays a popover containing the master view.

## Implementation

### Overview of iPathCase^KEGG^ capabilities

**Figure 2:**
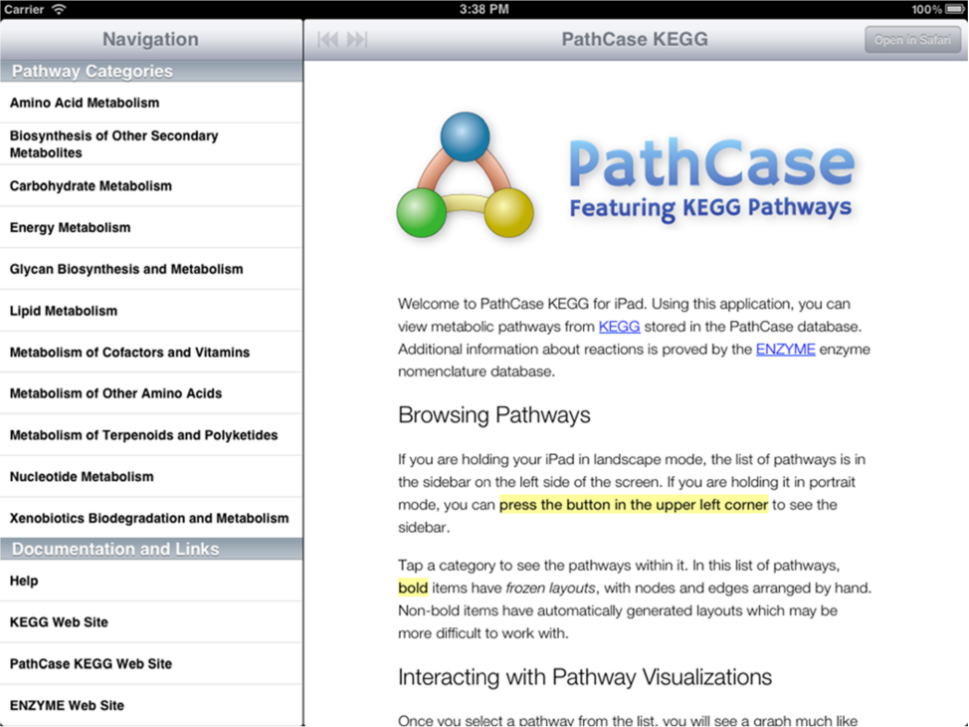
**Home screen for iPathCase** ^**KEGG**^ **.** List of pathway categories are displayed on the master view, along with a list of links at the bottom. Once clicked, web pages are displayed on the detail view.

**Figure 3:**
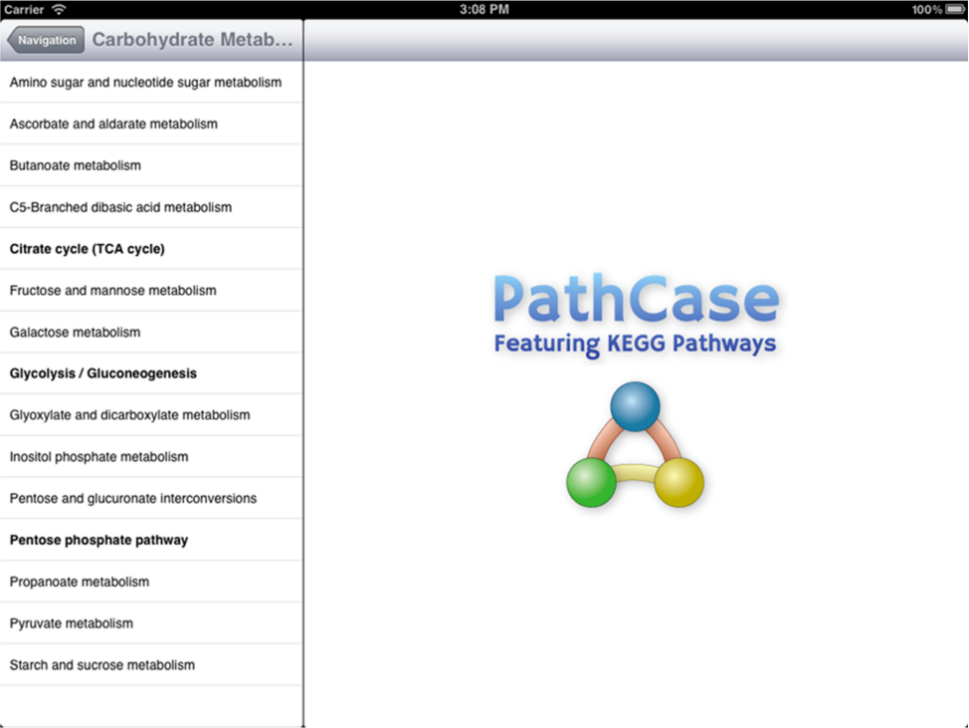
**List of carbohydrate metabolism pathways.** Master view shows the list of pathways in the carbohydrate metabolism category after it's selected from the list shown in Figure 6.

**Figure 4:**
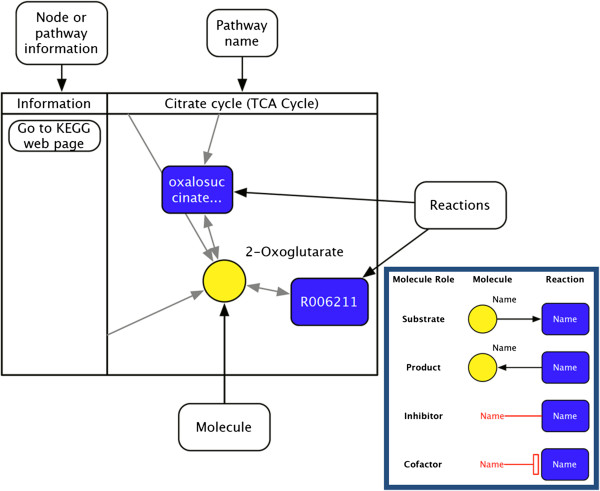
**Legend for pathway visualizations.** Figure shows possible interactions among molecules and reactions and labels each item shown in pathway visualizations. Rectangles represent reactions, circles represent molecules and edges show the direction of the reaction while associating molecules with reactions. Bidirectional edges represent reversible reactions.

**Figure 5:**
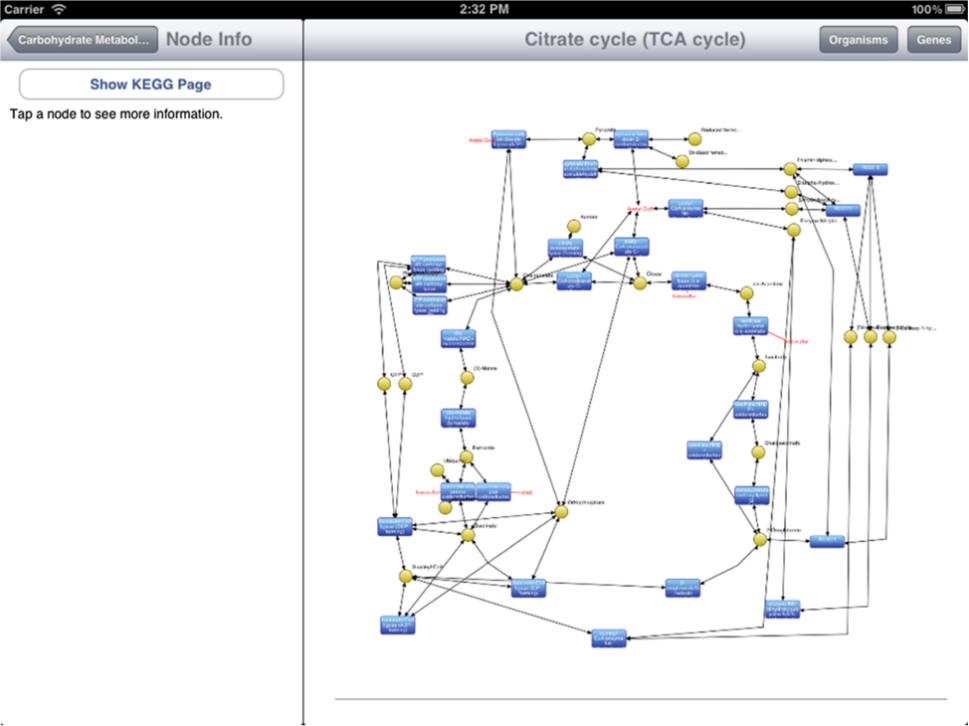
**Visualization of TCA Cycle.** TCA Cycle has been visualized in the detail view. Please see Figure 4 for the legend.

**Figure 6:**
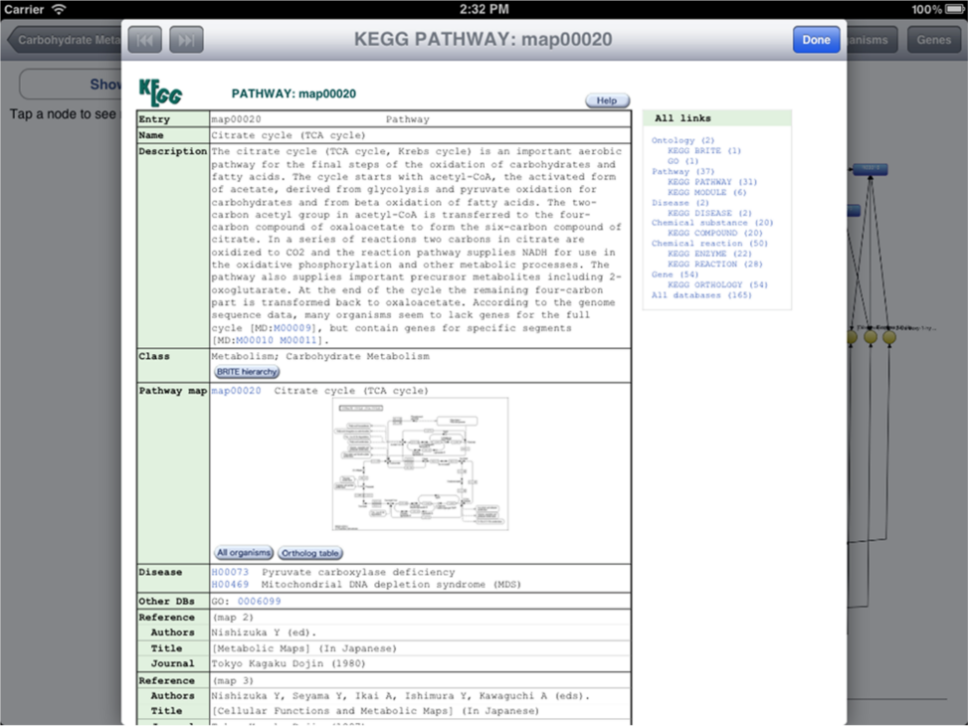
**KEGG web page for TCA Cycle.** Once the KEGG button is tapped, the web-page for the displayed pathway is opened as a popever. The KEGG web page for TCA Cycle is shown in this figure.

**Figure 7:**
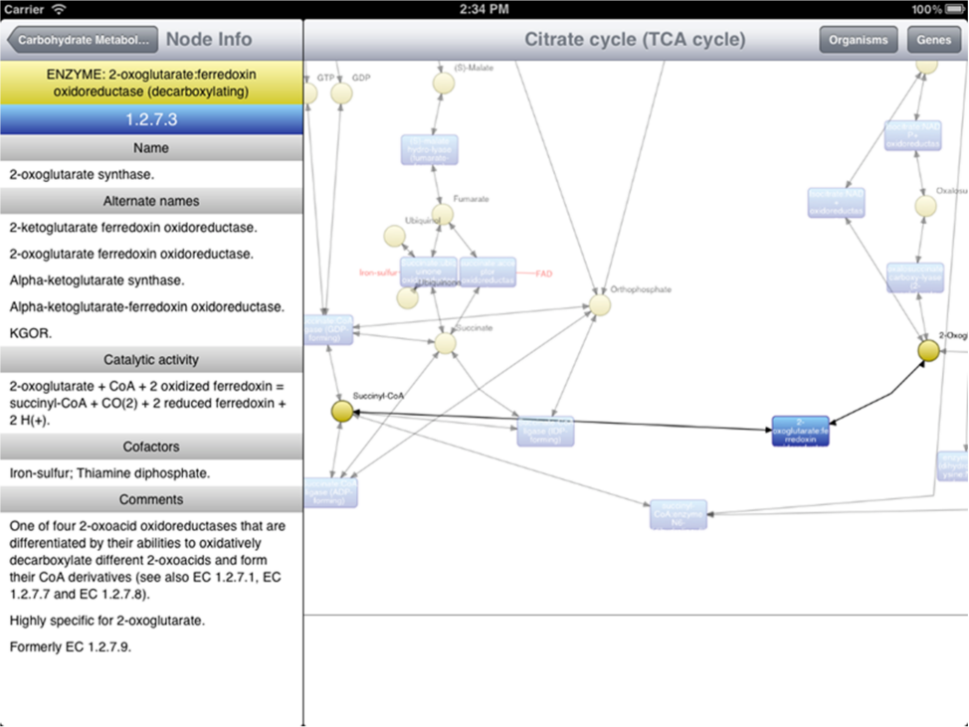
**Neighborhood of a tapped node is highlighted.** Once a node (reaction or molecule) is tapped in the visualization, all nodes within 1-hop distance are shown as is and the rest of the pathway is greyed out. This figure also illustrates the ENZYME database information related to the catalyzing enzyme of the tapped reaction. Here, ENZYME database information for *2-oxoglutarate.ferredoxin oxidoreductase* is displayed in the sidebar.

**Figure 8:**
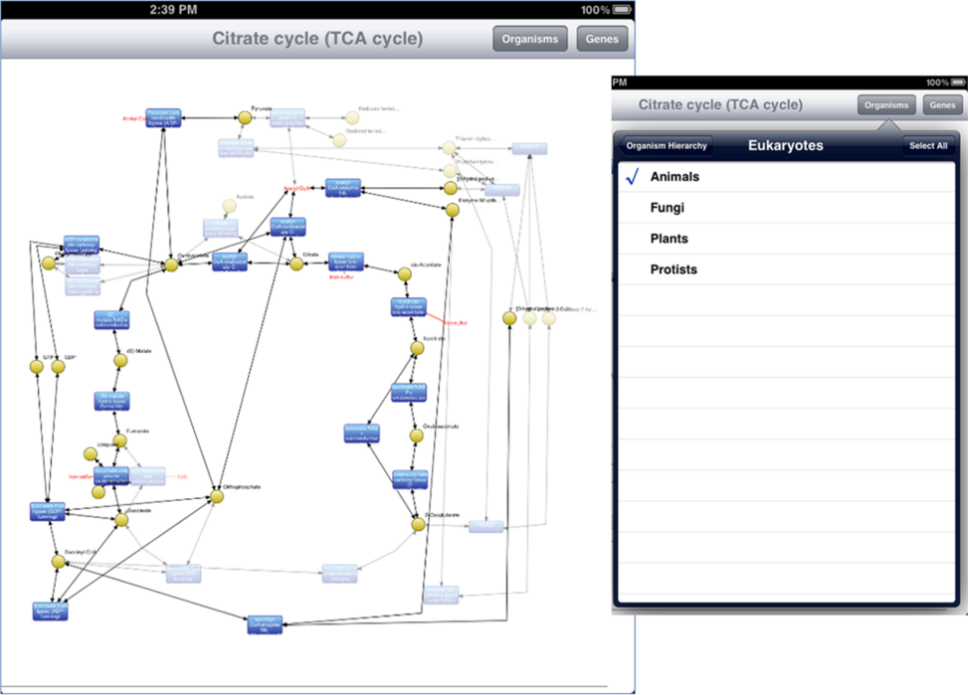
**Organism selection greys out the reactions not participating for selected organism.** Figure shows the reactions that participate in TCA Cycle pathway that only exist in “Animals”. Rest of the reactions are greyed out (on left). Tapping the button on top-right opens up the list of possible organisms that can be selected.

Here, we give a brief overview of iPathCase^KEGG^ interface to provide context to the implementation details that follow. iPathCase^KEGG^ divides the iPad screen into (i) a master interface (as a sidebar; master view) that provides navigation functionality and metadata for the currently selected object, and (ii) a details interface (detail view) that displays content chosen from the sidebar, such as documentation or a graph view. When the device is in the landscape (vertical) orientation, the master view is shown to the left of the detail view. In portrait (horizontal) orientation, it is accessed via a button in the upper left corner of the screen. This interface is described visually in Figure 1. The home screen of iPathCase^KEGG^ displays a list of metabolisms (i.e., pathway categories) and their documentation links in the sidebar as seen in Figure 2. Selecting a pathway category takes the user to a list of pathways. Figure 3 shows the list of pathways in Carbohydrate Metabolism category. Selecting a pathway from the list opens the graph view of that pathway in the detail view. A labeled diagram of an example visualization is shown in Figure 4 along with a legend for the different node types. The view for the TCA cycle is shown in Figure 5. So far, all of the displays have come from PathCase^KEGG^ database, and pathway layouts have come from GraphViz [14]. TCA cycle graph view can be panned and zoomed by touch. If no node is selected, the system displays a button which, when touched, accesses the KEGG web API, and shows the KEGG database web page for the selected pathway. One example page is shown in Figure 6. When a node is selected, any other node that is not connected to it by one hop is made transparent to make the node’s connections easier to see. Figure 7 shows an example of this behavior. “Organisms” button in the upper right corner triggers a popover, which allows the user to activate and deactivate organisms. If a reaction is not present in any activated organism, it is made partially transparent. The organism hierarchy menu is shown in Figure 8 on the right hand side. The graph view corresponding to the selection is shown on the left hand side.

In addition to displaying a node’s long description from PathCase^KEGG^, iPathCase^KEGG^ uses data from the ENZYME enzyme nomenclature database [12] to show more information for reactions for which EC numbers are available. ENZYME’s data comes from the recommendations of the Nomenclature Committee of the International Union of Biochemistry and Molecular Biology (IUBMB) [15]. An example of ENZYME information in the sidebar is shown in Figure 7 on the left hand side.

### Architecture

iPathCase^KEGG^’s architecture consists of a few well-defined components:

- *User interface objects* present things like the master-detail interface, lists of pathways, organism selection, etc.
- *Web service request classes* make HTTP requests to the PathCase^KEGG^ server, process the response, and notify other objects of the results.
- *Encapsulated components* provide specific functionalities such as reading the ENZYME database [12] and generating dynamic layouts with Graphviz [14].

**Figure 9:**
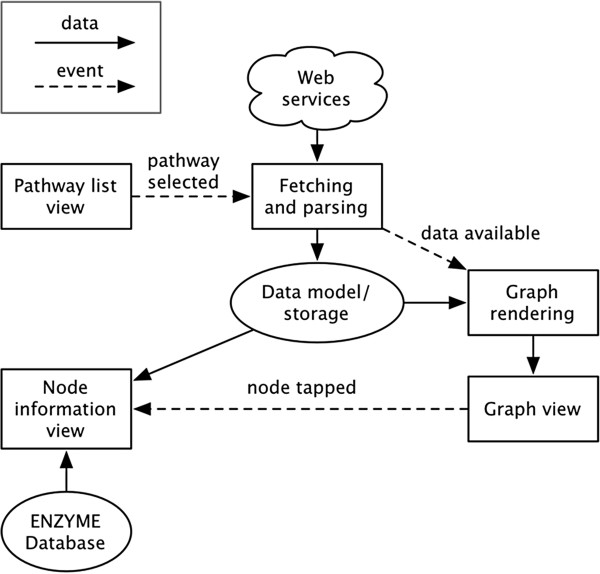
**High level diagram of the most important data flows and events in iPathCase-KEGG.**

Figure 9 presents a high level diagram of the most important data flows and events in iPathCase^KEGG^. Given the event-driven and nonlinear nature of GUI programming, it is more appropriate to describe events and notifications rather than straight line execution. The user begins at the pathway list view, which allows the selection of a pathway to view. Selecting a pathway initiates a call to the PathCase^KEGG^ web service (or a read from the local cache), the result of which is stored as an in-memory data structure. An event is then triggered to render the graph view from the stored data structure, and then display the rendered view itself. The graph view sends event notifications when nodes are tapped, which trigger the display of new information in the sidebar. This information comes from both the pathway data structure and a stored copy of the ENZYME database. There are pieces missing from the diagram in Figure 9, such as graph layout and the showing/hiding of organisms, but the essential pieces are present.

### Navigation user interface

**Figure 10:**
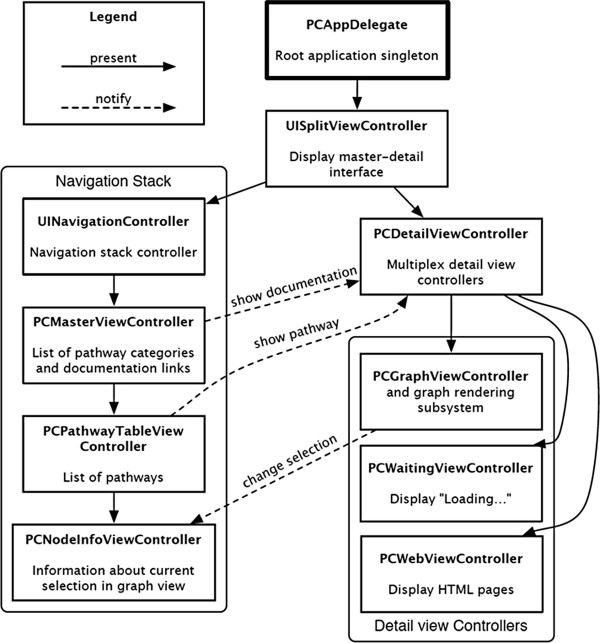
**Components of the user interface.** Figure details the individual components and their interactions for the user interface. Rectangles represent components, solid lines represent *present* edges that cause another controller to be displayed within that view controller, and dashed edges represent message passing between components.

Figure 10 shows a high-level representation of the user interface components of iPathCase^KEGG^. There are two kinds of edges in the diagram. Present edges are drawn when one view controller causes another to be displayed in its content area. For example, *UISplitViewController* shows the *PCMasterViewController* (via *UINavigationController*) in its sidebar area or popover, and the *PCDetailViewController* in the rest of its content area. Notify edges represent *notifications* that are sent between objects. A notification is an abstraction of message passing. An object may *register* for a notification of a certain name. When any object posts a notification with that name, the object that registered for the notification is passed a message that was specified at registration time. This message includes a *notification object*, which may contain additional data depending on the specific notification that was sent. This system allows objects to send and receive events without storing unnecessary references to other objects. A good example of notifications can be found in iPathCase^KEGG^. *PCMasterViewController*, a view controller included in Figure 10, shows a list of pathway categories and documentation links. Selecting a documentation link such as “KEGG Web Site” posts a notification called *PCPageViewRequested* that contains the URL of the corresponding web page, in this case http://www.kegg.com/.

*PCDetailViewController* responds to five different notifications, but only two are shown in Figure 10. The rest have to do with web services and pathway visualization interactions, which will be discussed later in this section. The remaining nodes in Figure 10, *PCPathwayTableViewController* and *PCNodeInfoViewController*, are displayed in the master content area. The master view makes use of a navigation stack. The top item on this stack is the *PCMasterViewController,* which pushes a *PCPathwayTableController* onto the stack when a pathway category is selected. When one of the pathways in that list is selected, a “show pathway” notification is posted, and a *PCNodeInfoViewController* is pushed onto the stack. This is the view controller that displays information for the current selection, so it responds to notifications sent by *PCGraphViewController* about changes in the user’s selection.

### Web services

iPathCase^KEGG^ does not download the entire pathways data in PathCase^KEGG^ database at once. HTTP requests are made as new data is needed, and the result is cached in the device’s internal storage. It has a separate class for each web service, named *PC < WebService > Fetcher*. iPathCase^KEGG^’s web service classes fire events to a central notification system when they have completed, and controller objects check to see if any new web services should be invoked due to dependencies being made available.

After the XML response has been parsed into one or more KEGG internal objects, these objects are not serialized. In other words, all objects generated by web services are built from the XML each time they are needed.

### Visualization

**Figure 11:**
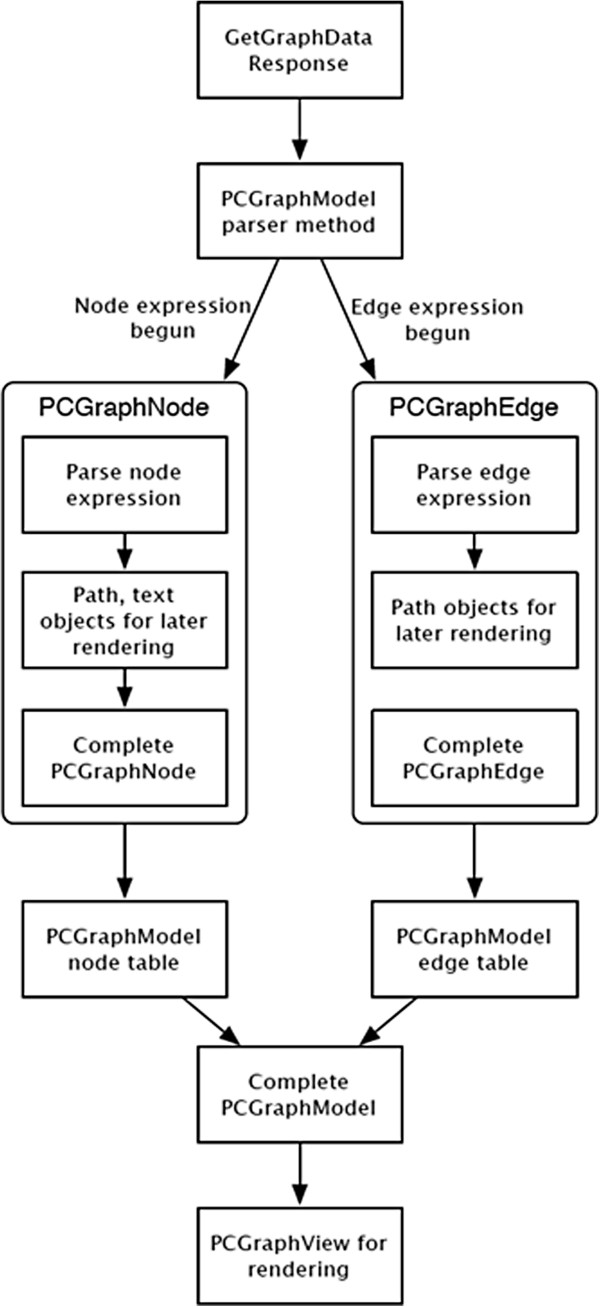
**Data flow from PathCase** ^**KEGG**^ **representation to final display format.** Figure shows the data flow for rendering a selected pathway. After the data is obtained from the server through the web service and parsed the nodes and edges are defined to form the graph model to be rendered. The representation is identical to Figure 2.

The pathway visualization data (graph data) is provided by the PathCase^KEGG^ web service *GetPathwayData*. Rather than calling the web service when the user requests to view a pathway, the web service responses for all pathways are included with the application itself in the form of text files. Each text file contains the HTTP response to a web request for the graph data for one pathway. Figure 11 shows the complete pipeline for converting data from *GetPathwayData*’s response format to the graph model in GML (Graph Markup Language) format. The GML is generated by the yFiles library [16]. iPathCase^KEGG^ graph representation has a *PCGraphNode* class to represent a graphical node and a *PCGraphEdge* class to represent a graphical edge. A *PCGraphModel* contains sets of these node and edge objects to represent single pathway visualization. In iPathCase^KEGG^, the node and edge objects contain no parsing code, but instead expose all graphical attributes as writeable properties. The responsibility for parsing a graph definition and building a graph model with the correct graphical attributes is moved to an external object that is not part of the graph rendering system. After data has been parsed, the graph is ready for rendering by a *PCGraphView,* which uses a *Core Animation* layer hierarchy to draw the graph efficiently. There are three layers in a *PCGraphView*. From top to bottom:

1. Text layer for drawing node labels. This layer contains one sub-layer for each node that renders the text for its label.
2. Node layer for drawing node shapes, and
3. Edge layer for drawing edges.

Once these layers are rendered, they are drawn to the screen by a scroll view, which takes care of translating and scaling them in response to user interaction.

### Life cycle of a pathway visualization

**Figure 12:**
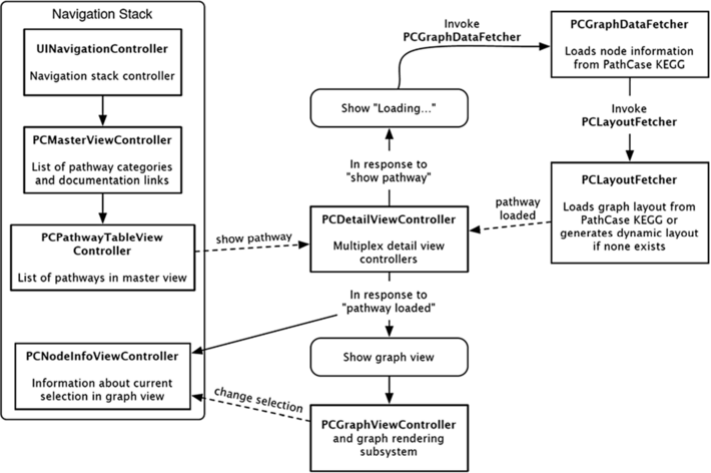
**Life cycle of a pathway visualization.** Figure shows the possible interactions among several components that contribute to pathway visualization. The representation is identical to Figure 2.

The loading and display of a pathway visualization requires interactions between several components of iPathCase^KEGG^. These interactions are illustrated in Figure 12. Loading is invoked by selecting a pathway from *PCPathwayTableViewController*. In response to the notification posted by this action, *PCDetailViewController* sets its content area to the “Loading…” screen, and invokes the *PCGraphDataFetcher* web service class to load the pathway’s information from the PathCase^KEGG^ web service *GetGraphData.* This class produces a set of *PCNodeInfo* objects that represent all aspects of the graph except node and edge positions. This set is given to a *PCLayoutFetcher* web service class, which attempts to download a layout for the graph from the PathCase^KEGG^ web service *RetrieveLayout*. If successful, it builds a *PCGraphModel* including a pre-drawn and manually beautified (so-called “frozen”) layout information using the information in the response. If not, it builds a *PCGraphModel* from the *PCNodeInfo* objects, and generates a dynamic layout. There are two advantages to using frozen layouts provided by *RetrieveLayout*. The first is that they are curated manually, and tend to look nicer as a result. The second is that, when a molecule is used in multiple reactions, each duplicate occurrence is drawn for each reaction, thereby eliminating many incoming and outgoing edges for a single common molecule such as water, or energy molecules ATP or ADP, and, hence, simplifying the visualization drastically. When a frozen layout is not available, iPathCase^KEGG^ generates dynamic layouts where a metabolite that is used in multiple reactions will appear only once, often resulting in scrambled layouts with one metabolite node having many incoming edges from many reactions. When the layout step is complete, *PCLayoutFetcher* posts a notification containing the generated *PCGraphModel* object. This notification is received by *PCDetailViewController*, which creates a *PCGraphViewController* and puts it in the detail view content area. It also pushes a *PCNodeInfoController* onto the master view navigation stack, which receives events from the *PCGraphViewController* about what it should display.

### Organism hierarchy

**Figure 13:**
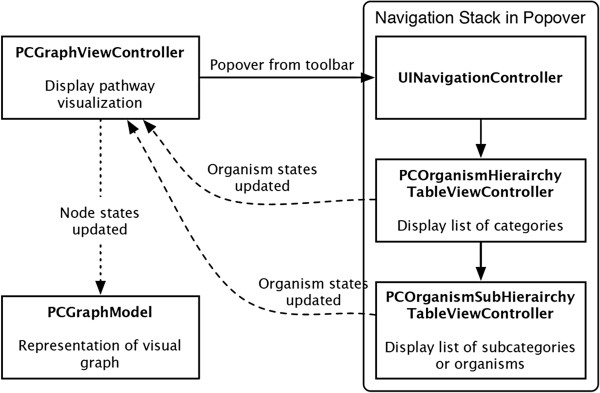
**Components of the organism hierarchy update process.** Figure shows the interactions among components to grey out parts of the network based the organism selection of the user. The representation is identical to Figure 2.

The iPathCase^KEGG^ pathway visualization includes the ability to select only a subset of organisms to display information from. If reaction *R* is present in organisms *A* and *B* but not *C*, and organisms *A* and *B* are disabled, then reaction *R* will be dimmed in the visualization. Next we briefly describe the process of displaying the organism hierarchy, shown in Figure 13. Selecting the toolbar button in the upper right corner of the visualization view causes *PCGraphViewController* to display a popover with a navigation stack. The first item on this stack is a *PCOrganismHierarchyTableViewController*, which presents a table view containing hierarchical categories of organisms. Tapping a row in the table view either pushes the subcategories of that category onto the navigation stack if the row represents a subcategory, or it toggles the enabled/disabled state of an organism if the row represents an organism. The set of enabled organisms is then posted as a notification that is received by the *PCGraphViewController*, which updates the *PCGraphModel* with the enabled/disabled state of each individual node based on the enabled/disabled state of the organisms.

### Dynamic layouts with graphviz

PathCase^KEGG^ contains human-curated frozen layouts for some pathways, but not all. In order to display the pathways without frozen layouts, iPathCase^KEGG^ uses the Graphviz library. Graphviz is a system which, among other things, provides graph layout and visualization functions [14]. It provides both command line and programmatic interfaces for entering, laying out, and rendering graphs. iPathCase^KEGG^ encapsulates the layout functionality of the Graphviz library in a class called *GVGraph*. An instance of this class represents a graph with nodes, a label for each node, and one directional edge that connect nodes.

### Reading the ENZYME database

The ENZYME database is provided in a flat text file format [12, 15]. This format is not acceptable for random seeking behavior, so it has been converted to a SQLite database [17] for more efficient access. SQLite is a minimal implementation of SQL intended to be used as a storage medium for single-instance applications [17]. iPathCase^KEGG^ uses the description, alternate name, catalytic activity, cofactor, and comments, ignoring the remaining fields.

## Results and discussion

Tablets have revolutionized the mouse and keyboard based interface with a mobile and a multi touch based interface. Many gestures such zoom-by-pinch or swipe to turn the page are very user-friendly. Not to mention the new retina display, iPad interface has been a breakthrough so far. As stated before, tablets have already been adopted as an educational tool. We foresee that tablets will keep attracting more attention and will be more widely used as prices drop; universities that distribute laptops to students will start to give tablets; classes will be held using tablets such as iPads; heavy hardcopy textbooks will be replaced by e-books, and students will be able to carry thousands of them in a single tablet device. For our application, KEGG is an important source for life sciences researchers, and for biology/biochemistry education as it maintains extensive biochemical pathways data. With the interactive visualization interface of iPathCase^KEGG^, users can interact with KEGG pathways using a multi-touch interface and gestures, which is a major improvement over the static visualizations provided by KEGG. We believe that iPathCase^KEGG^ is useful for (i) students who use KEGG to learn metabolic networks, (ii) instructors that teach material using a visual tool and (iii) researchers who would like to employ metabolic pathways in their research.

### Comparison with Web-based PathCase^KEGG^

Table 1 compares functionalities available in the interfaces of the web-based PathCase^KEGG^ system and the iPathCase^KEGG^ app. Most of the functionalities listed are self-explanatory, and, next, we discuss only a few of the functionalities listed in Table 1.

**Table 1:** **Comparison of web-based PathCase** ^**KEGG**^ **and iPathCase** ^**KEGG**^ **interfaces**

	Web-based	iPad
Molecule and reaction visualization	Yes	Yes
Navigation method	Four mouse-based navigation tools	Pan with one finger, zoom with two
Greying out reactions that are not present in the selected organism set	Yes	Yes
Moving nodes to rearrange the layout	Yes	Yes
Dynamic layout when no frozen layout is present	Yes	Yes
Saving layouts for future recall	Yes	No
Lists of processes, connected pathways, pathway annotations, and pathway-related queries in tabular form	Yes	No
Identifying the location of the gene producing the catalyzing enzyme	Yes	No
Hiding common molecules	Yes	No
Display of data from ENZYME database	No	Yes
Display of data from KEGG web site	No	Yes

The functionality “*Greying out reactions that are not present in the selected organism set*” is available in both PathCase^KEGG^ and iPathCase^KEGG^, which allows the user to focus on only those reactions of the pathway available in the chosen organism set. The functionality “*Moving nodes to rearrange the layout*” of the pathway at hand is also available in both interfaces. As it turns out, biochemists always would like to have the same layouts for pathways, and automated layouts may need to be manually edited by some users; hence this functionality is considered essential for life sciences researchers. Also, this functionality gives the user the flexibility of changing the layouts based on their specific needs, i.e., emphasizing a certain part of a given pathway.

The functionality "*Identifying the location of the gene producing the catalyzing enzyme*" allows pathway analysis in terms of the locations of genes producing catalyzing enzymes of different reactions. This functionality can be viewed as a higher level pathway analysis functionality, and is not provided in iPathCase^KEGG^.

*Common molecules* are those molecules that participate in many reactions, i.e., CO_2_, ATP, ADP, NAD+, NADP+, NH4+, NADPH, NH3, NADH, Oxygen, CoA, H+, H_2_O, H_2_O_2_. Common molecules can also be regarded as social network “hubs” of pathways. PathCase^KEGG^ allows users to “hide” common molecules when multiple pathways are drawn together as it reduces the visualization complexity of pathways. This feature is also not provided in iPathCase^KEGG^ since, due to its small screen size, we have opted not to allow users of iPathCase^KEGG^ to visualize multiple pathways together as one sub-network of the whole metabolic network.

### Technical challenges and possible improvements

The biggest challenge of the project was controlling the passage of information from the PathCase servers to the iPad application, both in how the HTTP requests were made and how the data was translated into a form usable by the rest of the application. The data is requested from the PathCase^KEGG^ server in a much more piecemeal way. The data for each pathway is encapsulated entirely in a single request; so there is no need for complex dependency management or even data storage above and beyond simple HTTP request caching. The data models, HTTP requests, and parsing are cleanly separated.

When the project started, we underestimated the software system complexity involved in iPathCase^KEGG^ (as well as other iPathCase systems). In terms of software architecture, in the future, the system components that download the data, parse it, build model objects, as well as various controls should be re-factored to separate each of these tasks explicitly. Additionally, there should be a more formal data dependency tracking system to control the bulk download of the web service data. It is common to have HTTP requests for web services *A*, *B*, and *C* rely on data from request *X*, so there should be a formalized dependency graph that tracks this information.

iPathCase^KEGG^’s biggest technical issue is a noticeable hang when selecting a large pathway to view, due to the HTTP requests and responses that involve creation and transfer of large (2–5 megabytes) XML documents, sometime with large frozen layout information, as well as dynamic layout creation delays due to the use of Graphviz. Caching pathways a priori has mostly, but not fully, eliminated this problem. However, when a new or revised pathway in the PathCase^KEGG^ system is encountered and it needs a dynamic layout generation, the system takes significant amounts of time (ranging from 30 to100 seconds) to request and receive the new/revised pathway, parse and produce a new layout, and display it. A delay of this magnitude sometimes leads users into thinking that the app has crashed, which is not acceptable in an online visualization environment. More effort should be spent for both (a) lazy processing and downloads of new pathways (perhaps as part of “software updates” to the app which are really data updates), and (b) moving processing to a separate thread to allow the main user interface to remain responsive.

Yet another technical challenge was rendering large graphs efficiently on a device with limited memory and processing power. Fortunately, the iOS application frameworks made pathway rendering possible, though various low-level optimizations were required at different stages of development.

Finally, similar to graph rendering, dynamic graph layouts accounted for a considerable amount of development time, despite the convenience of the Graphviz library to provide layouts on an as-needed basis.

## Conclusions

iPathCase^KEGG^ has been a successful exercise in extending the PathCase architecture and philosophy to new devices and new users. It is one thing to design a web interface and server backend in tandem, and another to apply the server’s web service interface to an entirely new set of applications. iPathCase^KEGG^ shows that PathCase’s architecture serves the purpose for which it was built: to provide a platform for analysis and reference tools on top of multiple data sources for pathways. iPathCase^KEGG^ :

- Allows browsing of all KEGG pathways,
- Links reactions with the external ENZYME database, and
- Allows access to the KEGG web page corresponding to any pathway.

The application adapts the basic PathCase pathway visualization interface concepts for a touch screen, specifically making heavy use of iOS idioms. This adaptation and iPathCase^SMDA^[18], another PathCase iPad application already at the Apple App Store, provide insight about how pathway visualizations can be improved across all forms of input and display.

iPathCase^KEGG^ opens up many exciting possibilities for the use of tablets in educational and research environments. The best thing that can be done to make these possibilities become realities is to perform studies and have discussions with practicing life scientists and students. Formal and informal feedback should be gathered from possible users to determine the most effective future improvements. Even their simple presence in the iOS online store should provide helpful feedback (positive or negative) that will affect the development of both applications.

Interactive visualizations of complex biological data are no longer limited to immobile desktop workstations or cumbersome laptops; the iPad is even less intrusive than a physical textbook and can display information in new and interesting ways. iPathCase^KEGG^ brings interactive pathway visualizations, reference materials, and even research tools to desks, meetings, and couches.

## Availability and requirements

- **Project name:** iPathCase^KEGG^
- **Project download source:** Apple App Store
- **Operating system:** iOS 5.
- **Programming language:** Objective-C.
- **Other requirements:** iPad2 or newer.
